# A century (1919-2019) of academic midwifery in Iran: From traditional midwives to PhD graduates

**DOI:** 10.18332/ejm/110065

**Published:** 2019-06-20

**Authors:** Sevil Hakimi

**Affiliations:** 1 School of Nursing and Midwifery, Research Center of Psychiatry and Behavioral Sciences, Tabriz University of Medical Science, Tabriz, Iran

**Keywords:** midwifery, history

The history of midwifery in Iran, in the form of academic education, is now 100 years old. Before this, there was no classic midwifery education; only traditional midwives had this task, and their lack of knowledge led to high maternal and neonatal mortality. For the first time in 1919^1^, ten students of the Franco–Persian school (a special school for girls) in Tehran were selected to train as midwives through hospital-based training. This was the basis of academic midwifery education in Iran. The first Midwifery school officially opened in 1926^2^, while in 1929 the midwifery school became affiliated with the faculty of medicine. The students of this school were obligated to possess primary school certification (5 years of formal education). The duration of education of midwifery at that time was 3 years^3^.

In 1955, nursing and midwifery training were merged, and the title of ‘registered nurse-midwife’ was awarded following 3 years nursing and 12–18 months of midwifery courses. From 1970 until 1985, due to a significant lack of midwives in the work force, a two-year associate degree of midwifery was initiated, after obtaining a high school diploma.

For the first time, in 1986, a Bachelor of Midwifery program was launched as an independent major in a few national universities, and this was a turning point for academic midwifery in Iran. Four years later, a postgraduate program (Master’s degree in Midwifery) was started, while in 2006, the first PhD program in ‘reproductive health’ was launched in five public universities.

Currently, more than 40 public (governmental) universities have a Bachelor of Midwifery program and each year 700–800 students enter such a program. Ten universities have a Master of Midwifery program with five orientations: midwifery education, maternal and child health, community-based midwifery, forensic midwifery, and reproductive health. Annually, 70–80 students are accepted in a Master of Midwifery program. At present, two doctorate programs are also offered (PhD in Midwifery, and PhD in Reproductive Health) in eight universities in Iran, with 25–30 students entering this program every year. Additionally, a Master of Counselling in Midwifery program has been designed and established since 2014 at six universities in Iran .

The goals of the undergraduate and postgraduate midwifery programs in Iran are: improvement of maternal and newborn health, training professional midwives to train students for the Masters courses in the universities and hospitals, and empowerment of midwives in research and policy making.

Since 1995, the Midwifery Office in the Ministry of Health (MOH) was separated from the Nursing Office, and from 2004, this office was merged with the Maternal Health Bureau of MOH. This office closely collaborates with international agencies, including WHO, UNICEF, and UNFPA^3^.

For the past 45 years, midwives have been able to establish private offices with membership in the Iranian Medical Council and privately visit patients as part of their job responsibilities. Midwifery job responsibilities have been developed and are regularly revised by the Ministry of Health^4^.

One hundred years after the start of academic education in midwifery, more than 30000 midwives (with Bachelor’s, Master’s and PhD degrees in midwifery) work in the Iranian public health system. In recent years, Iran has achieved remarkable progress in maternal and newborn health. From 1990 until 2015, Iran reduced MMR (Maternal Mortality Ratio) by more than 75%, as determined by WHO^5^. Midwives have an incredible role in the reduction of maternal and neonatal mortality and morbidity, increasing use of contraception, decreasing unintended pregnancies, and increasing the exclusive breastfeeding rate. However, there are several challenges in this field. The overall CS (cesarean section) rate in Iran is near 50%^6^. Respectful maternal care, which is strongly recommended by WHO^7^, has yet to be completely implemented in the country. Family center care in the maternal and newborn field has not been integrated into the routine maternal care, and for this to take place more midwives are needed in Iran. WHO has estimated the nurse-midwife workforce in Iran to be 18.1 per 100000 population.

Midwifery job satisfaction is not ideal, as regional research shows that job satisfaction among midwives is at an average level^8,9^. Improving job satisfaction, job security, and providing funding for hiring as well as training midwives by the health system are necessary so as to see Midwifery in Iran flourish also in the next century.
